# Reassessing Reported Propofol Allergy in a Patient Requiring Jet Ventilation: A Case Report

**DOI:** 10.7759/cureus.112664

**Published:** 2026-07-14

**Authors:** Autumn Smith, Peni Daly, Maisie Jackson

**Affiliations:** 1 Anesthesiology, Baylor College of Medicine, Houston, USA

**Keywords:** jet ventilation, perioperative anaphylaxis, propofol allergy, shared airway surgery, skin prick testing

## Abstract

A 39-year-old man with severe subglottic stenosis required jet ventilation and total intravenous anesthesia for high-risk airway surgery. However, the patient reported a propofol allergy characterized by facial and tongue swelling, raising concern for prior anaphylaxis. Because alternative anesthetic techniques were considered suboptimal, Allergy and Immunology performed skin prick and intradermal testing to propofol, both of which were negative. Following an intraoperative test dose, propofol-based total intravenous anesthesia (TIVA) was administered safely without complication. This case highlights the importance of systematic evaluation of reported anesthetic allergies in facilitating optimal perioperative management in patients undergoing complex surgeries.

## Introduction

Propofol is one of the most widely used intravenous anesthetic agents due to its rapid onset and favorable recovery profile. Although generally considered safe, perioperative hypersensitivity reactions, including anaphylaxis, have been reported. The estimated incidence of perioperative anaphylaxis ranges from one in 10,000 to one in 20,000 anesthetic procedures, with hypnotic agents such as propofol representing an uncommon cause [1]. Despite its rarity, reported propofol-associated hypersensitivity presents diagnostic challenges due to variable clinical manifestations, incomplete documentation, and limitations of allergy testing. 

Reported allergic reactions are frequently not investigated and are instead managed by avoidance of the suspected offending agent. Fortunately, alternative medications exist for nearly all drugs commonly used in the perioperative setting. Designing an anesthetic plan that balances efficacy with minimization of adverse effects is a central challenge in anesthetic practice. In many situations, creative combinations of alternative agents can provide safe and effective anesthesia. However, there are specific clinical scenarios in which a particular medication is clearly superior. In these cases, a reported but uninvestigated allergy may become not only an inconvenience for the care team, but also a potential barrier to optimal patient care.

This case of a high-risk airway surgery requiring jet ventilation underscores the importance of thorough documentation, systematic evaluation, and appropriate allergy testing in patients with suspected anesthetic hypersensitivity reactions. Written Health Insurance Portability and Accountability Act (HIPAA) authorization was obtained from the patient, including permission to publish relevant clinical details and imaging.

## Case presentation

A 39-year-old man with severe subglottic stenosis following prolonged intubation was transferred from an outside hospital for laser ablation and balloon dilation of the trachea by the otolaryngology service. Given the critical airway narrowing, the surgical and anesthesia teams planned direct laryngoscopy with jet ventilation requiring total intravenous anesthesia (TIVA).

The patient’s medical history was significant for end-stage renal disease, coronary artery disease status post coronary artery bypass grafting, congestive heart failure (CHF), obstructive sleep apnea, and morbid obesity. During a prior hospitalization for a CHF exacerbation, the patient required prolonged intubation for seven days, which was believed to be the cause of his subglottic stenosis. Following extubation and recovery, he continued to experience noisy breathing and exertional dyspnea. Subsequent evaluation demonstrated tracheal stenosis with a minimal airway diameter of 0.7 cm.

During his preoperative anesthesia evaluation, the patient reported a severe allergy to propofol. When questioned further, he stated he had been told he experienced “facial and tongue swelling” after receiving the medication. All prior medical care had occurred at hospitals in a neighboring state. A detailed review of available records revealed that the reaction was first documented while the patient was in the intensive care unit. Multiple notes described facial and tongue swelling; however, there was no clear documentation of other associated findings such as hypotension, bronchospasm, urticaria, or rash. Additionally, there was no evidence that serum tryptase, immunoglobulin levels, or complement studies were obtained during the suspected reaction. Likewise, no formal allergy evaluation, including skin prick testing, intradermal testing, or basophil activation testing, had been performed. Given the limited and nonspecific documentation, the nature of the reported reaction remained unclear.

Despite the lack of formal allergy testing, the patient's prior reaction was documented as a propofol allergy in his medical records. Since this documented reaction raised concern for possible anaphylaxis, alternative anesthetic strategies were considered, including remifentanil infusion with intermittent etomidate boluses, dexmedetomidine-based anesthesia, or ketamine-based anesthesia. However, the authors opined that none of these approaches offered the same balance of efficacy and predictable operating conditions as propofol-based TIVA. The possibility of using an endotracheal tube with inhaled anesthetics rather than jet ventilation was also discussed with the otolaryngology team. However, the smallest endotracheal tube available at our adult hospital was a 5.0-mm tube with an outer diameter of 8.0 mm, larger than the patient’s stenotic tracheal diameter.

As the surgery was not emergent, Allergy and Immunology was consulted to evaluate the possibility of propofol allergy testing. They recommended skin prick testing and intradermal testing for propofol. Undiluted propofol was used for skin prick testing, and a 1:10 dilution was used for intradermal testing. Saline and histamine were used as negative and positive controls, respectively. After 30 minutes, an appropriate response to histamine was observed, with no reaction to either propofol or saline, indicating no evidence of immunoglobulin E (IgE)-mediated hypersensitivity. Allergy and Immunology subsequently recommended administration of a test dose of propofol in the operating room, followed by a 15-minute observation period prior to full induction.

The recommended test dose of 2 cc propofol was given in the operating room, and after no evidence of hypersensitivity after 15 minutes while fully monitoring the patient, he received a standard induction dose of propofol and was subsequently maintained on propofol and remifentanil infusions throughout the procedure. Jet ventilation was successfully utilized while the stenotic segment was ablated and dilated. Both the intraoperative and postoperative courses were uneventful, with stable hemodynamics and no evidence of hypersensitivity.

## Discussion

True propofol anaphylaxis is rare, with an incidence estimated at approximately 0.06 per 10,000 anesthetics in one large pediatric series [2]. However, in clinical practice, reported or even suspected propofol allergy is frequently managed by avoidance and substitution with alternative anesthetic agents, often without difficulty. In most cases, induction with etomidate followed by maintenance with volatile anesthesia or non-propofol total intravenous anesthesia provides acceptable conditions.

This case was unusual in that the need for jet ventilation in the setting of severe subglottic stenosis precluded the use of inhaled anesthetic techniques, as the operative setup required an open airway shared with the surgical field. Several alternative total intravenous anesthetic strategies were considered, including remifentanil-based infusions with adjunctive agents such as dexmedetomidine or ketamine; however, each was felt to have limitations in providing the degree of immobility, titratable hypnosis, and rapid intraoperative adjustability required for laser airway surgery with jet ventilation.

Etomidate was also considered as a potential hypnotic agent given its hemodynamic stability in high-risk patients. Although continuous etomidate infusions have been described, their use went out of favor in the ICU setting after reports of sustained adrenal suppression after prolonged infusions [3]. One randomized trial did compare etomidate TIVA vs. propofol TIVA and found no increase in perioperative mortality with etomidate, along with less hypotension during surgery [4]. However, even with short infusions, cortisol and aldosterone levels were decreased postoperatively and up to three days following surgery. Considering the potential risks and the limited evidence supporting its use, the care team concluded that etomidate should be reserved as a second-line agent, to be used only if the reported propofol allergy could not be excluded. These considerations underscored the importance of formally re-evaluating the reported propofol allergy, ultimately allowing the use of propofol-based total intravenous anesthesia, a more predictable, well-established, and extensively validated anesthetic technique.

When perioperative hypersensitivity is suspected, a stepwise diagnostic approach is recommended [1]. During the acute phase, serum tryptase measurement can help confirm mast cell degranulation and should ideally be obtained within 30 minutes to two hours after symptom onset [5]. A normal tryptase level does not exclude hypersensitivity, as the reported sensitivity is approximately 64%; however, it remains a valuable diagnostic tool when available [6]. In this case, no evidence of acute-phase allergy evaluation could be identified.

The next step in evaluation is skin testing, typically performed four to six weeks after the suspected reaction to minimize the risk of false-negative results related to mast cell mediator depletion. Skin testing remains the gold standard for identifying IgE-mediated reactions [1]. Skin prick testing (SPT) and intradermal testing (IDT) are designed to detect immediate Type I hypersensitivity reactions mediated by allergen-specific IgE. These mechanisms underlie common allergic conditions including allergic rhinitis, asthma, food allergy, insect venom allergy, and drug hypersensitivity reactions. A positive skin test is generally defined as a wheal measuring at least three mm greater than the control.

For propofol specifically, sequential skin prick and intradermal testing has been reported to have a negative predictive value of 96-100% [5]. Furthermore, even after negative skin testing, a graded dose challenge may be used to definitively exclude clinically significant IgE-mediated hypersensitivity [5,6]. This stepwise strategy consisting of serum tryptase measurement, delayed skin testing, and graded drug challenge can effectively identify or exclude culprit agents in most perioperative hypersensitivity reactions. If both skin testing and graded challenge are negative, the medication can generally be safely removed from the patient’s allergy list [7].

Historically, concern regarding propofol allergy has been amplified by its formulation containing egg lecithin and soybean oil. However, multiple studies have demonstrated no relationship between allergy to egg, soy, or peanut and hypersensitivity to propofol [2,8]. Consequently, food allergy history alone should not prompt avoidance of propofol or routine allergy testing. In contrast, a history suggestive of prior perioperative hypersensitivity to anesthetic agents warrants formal evaluation before future exposure.

## Conclusions

This case highlights how systematic perioperative allergy evaluation can facilitate the use of a preferred anesthetic technique in a situation where alternative approaches may compromise procedural safety or efficacy. In patients with complex airway pathology requiring jet ventilation, propofol-based TIVA represents a uniquely well-suited anesthetic strategy, and an unverified allergy history should not preclude its consideration when alternatives are suboptimal. Rather than defaulting to avoidance, clinicians should recognize that reported drug allergies, particularly those lacking objective documentation, acute-phase biomarkers, or formal allergy evaluation, warrant systematic re-investigation when the clinical stakes are high. Collaboration between anesthesiology and allergy and immunology was central to this outcome, enabling a structured, evidence-based approach that included skin prick testing, intradermal testing, and an intraoperative graded challenge. The negative predictive value of sequential skin testing for propofol is well-established and, when combined with a supervised test dose, can provide sufficient reassurance to proceed safely. This case therefore serves as a model for interdisciplinary perioperative allergy evaluation and underscores the broader principle that a documented allergy should be a prompt for investigation rather than an automatic barrier to optimal care.
